# Toxoplasmosis outbreak in São Paulo, Brazil: Epidemiology and visual outcome

**DOI:** 10.5935/0004-2749.2022-0374

**Published:** 2024-03-05

**Authors:** Luciana Peixoto Santos Finamor, Geraldine Madalosso, José Eduardo Levi, Yuslay Fernández Zamora, Gabriela Akemi Kamioka, Paula Marinho, Heloisa Nascimento, Cristina Muccioli, Rubens Belfort Jr

**Affiliations:** 1 Department of Ophthalmology and Visual Science, Universidade Federal de São Paulo, São Paulo, SP, Brazil; 2 Divisão de Vigilância Epidemiológica, Vigilância Sanitária, São Paulo, SP, Brazil; 3 DASA Laboratories, São Paulo, SP, Brazil; 4 Instituto Paulista de Estudos e Pesquisa em Oftalmologia, São Paulo, SP, Brazil

**Keywords:** Toxoplasmosis/etiology, Food parasitology, Water/parasitology, Uveitis, posterior/parasitology, Chorioretinitis/parasitology, Visual acuity, Disease outbreaks, Eye manifestations, Humans

## Abstract

**Purpose:**

To describe a 2019 acute toxoplasmosis outbreak in the city of São Paulo, Brazil, and to evaluate the laboratory serological profile for toxoplasmosis for three consecutive years. The ophthalmological manifestations of the patients involved in the outbreak were also studied.

**Methods:**

A cross-sectional descriptive study of a toxoplasmosis outbreak in São Paulo, Brazil, between February and May 2019. Epidemiological data were described, as were the observed ocular manifestations. As part of this study the number of patients with positive IgM toxoplasmosis serology was obtained from a large laboratory network (DASA) for three consecutive years, including the year of the outbreak (2018, 2019, 2020).

**Results:**

Eighty-three individuals were identified in the outbreak and two clusters were studied. The clinical picture of at least 77% of the patients, the epidemiological analysis, and the short incubation period (5-8 days) suggested contamination by oocysts. Serological laboratory data analysis revealed an increase of positive toxoplasmosis IgM in 2019 of 73% compared to the previous year. Ophthalmological examination revealed that at least 4.8% of the patients developed toxoplasmic retinochoroiditis, none of whom had been treated during the acute systemic disease.

**Conclusion:**

Our findings indicate vegetable contamination as the possible source of this outbreak, a high prevalence of toxoplasmosis in São Paulo during the outbreak period, and a drop in the number of tests during the COVID-19 pandemic. Retinochoroiditis was observed in at least 4.8% of the cases. We confirm the need to implement effective means for the prevention, diagnosis, and treatment of the disease. This may involve raising awareness among the population of the importance of vegetable hygiene, and improved quality control of food and water.

## INTRODUCTION

Toxoplasmosis is an infection caused by the intra-cellular parasite *Toxoplasma gondii.* At least one third of the world’s population is infected by the parasite. Ocular infections by the parasite are common in Brazil^(1)^ where toxoplasmosis prevalence varies from 10 to 90% of the adult population^(2)^.

Epidemiological surveys over decades have shown wide differences in the prevalence of *T. gondii* infection globally^(3)^. The ingestion of tissue cysts in raw or under-cooked meat, or of sporulated oocysts in contaminated food or water are the modes of transmission in humans. Oocysts have been implicated in several toxoplasmosis outbreaks due to environmental contamination. Contamination of drinking water was associated with large outbreaks in Canada^(4)^ and Brazil^(5)^, however, raw meat is still the main source of contamination in most cases.

In February 2019, the *Epidemiological Surveillance of Foodborne Diseases from São Paulo, Brazil,* observed an increase in the number of reported cases of acute toxoplasmosis in the city of São Paulo. In the same month, there was also an increase in positive toxoplasmosis tests through laboratory surveillance (Madalosso, Geraldine Centro de Vigilânca Epidemiológica, Departamento Estadual de Saúde, São Paulo, Brazil, 2019 Feb 27. Personal communication).

This study aimed to describe an outbreak characterized by two clusters of acute toxoplasmosis in the city of São Paulo, Brazil, from February to May, 2019 and compare the serologic profile for toxoplasmosis during the outbreak with previous and consecutive years. We also aimed to explain this outbreak in such a large city. Ophthalmological outcomes for 14 months after the outbreak were also studied.

## METHODS

The outbreak’s epidemiological investigation was conducted by the São Paulo City Health Department, *Coordenadoria de Vigilância em Saúde* (COVISA) from February to May 2019. The number of patients with positive IgM serology for toxoplasmosis for the years 2018, 2019, and 2020 was obtained from a large laboratory network (DASA).

Two clusters were confirmed: one in an Arabic food restaurant (A) and the other in a barbecue party (B). Patients were investigated regarding food intake, and for systemic and ocular clinical involvement. Laboratory work-up included toxoplasmosis serology and tests for other infectious and non-infectious possible causes. All individuals attending the events were invited to have an eye examination that included visual acuity, ocular biomicroscopy, tonometry, and indirect ophthalmoscopy. Fundus photography and optical coherence tomography (OCT) were performed when abnormalities were discovered. Patients were instructed to return if they developed ocular symptoms.

For the statistical analyses, Fisher’s test was used for categorical variables and logistic regression for the non-categorical variable (age).

The institutional review board of the Federal University of São Paulo approved the protocol (IRB 3.523.706). All patients provided informed consent. The study was performed according to the tenets of the Declaration of Helsinki and complied with the Health Insurance Portability and Accountability Act of 1996.

## RESULTS

### Health surveillance

Epidemiological data were obtained from the outbreak epidemiological investigation report prepared by the São Paulo City Health Department. Between February and May 2019, the São Paulo health surveillance department (COVISA) identified two clusters involving 83 individuals from two events who were positive for toxoplasmosis IgM.

### Laboratory data

Among the 26,752 serum samples tested during the outbreak, an IgM seropositivity of 3.1% (n=816) was observed. Seropositivity was greater in the period of the outbreak, with a statistically significant difference (p<0.01).

A higher seroprevalence was observed in the tested males during the outbreak (7.0%) which represents an increase of 122% in the number of cases in males. In 2020, IgM positivity in males was 1.4%, the lowest of the studied period.

Regarding age, the greatest increase of positive cases during the outbreak was in individuals between 45 and 60 years old. In women, the most relevant difference occurred in the age group between 45 and 55 years.

In 2019, the year of the outbreak, we had an increase of 8.7% in testing. In 2020, during the COVID-19 pandemic, we observed a reduction of 15.7% compared to the previous year. The prevalence of positive cases was 1.8% in 2018, 2.8% in 2019, and 1.4% in 2020 (Table 1). This difference was significant (p<0.01).

**Table 1:** Laboratory Data: Distribution of toxoplasmosis serology tests and percentage of IgM positive serology during the months of the outbreak (February-May) and from January-December 2018, 2019, and 2020

	Feb-May				Jan-Dec			
Number of cases tested	2018 (n=22.609)	2019 (n=26.752)	2020 (n=18.864)	Total (n=68.22)	2018 (n=62.061)	2019 (n=67.464)	2020 (n=56.871)	Total (n=186.396)
Women	19.990 (88.4%)	23.902 (89.3%)	15.674 (83.1%)	59.566 (87.3%)	54.995 (88.6%)	59.358 (88.0%)	50.884 (89.5%)	165.237 (88.6%)
Ig M+	434 (1.92%)	816 (3.1%)	258 (1.37%)	1.508 (2.21%)	1.104 (1.78%)	1.912 (2.83 %)	779 (1.37%)	3.795 (2.04%)
Women Ig M+	352 (1.76%)	615 (2.57%)	213 (1.35%)	1.180 (1.98%)	840 (1.35%)	1.572 (2.33%)	606 (1.06 %)	3.018 (1.62%)
Men Ig M+	82 (3.13%)	201 (7.0%)	45 (1.41%)	328 (3.79%)	264 (3.73%)	340 (4.19%)	173 (2.88%)	777 (3.67%)

During the evaluated period, 3,795 cases with IgM positivity for toxoplasmosis were noted, 79.5% were in females. The distribution of the cases in men and women during the 3 years is presented in table 1.

**Figure 1:**
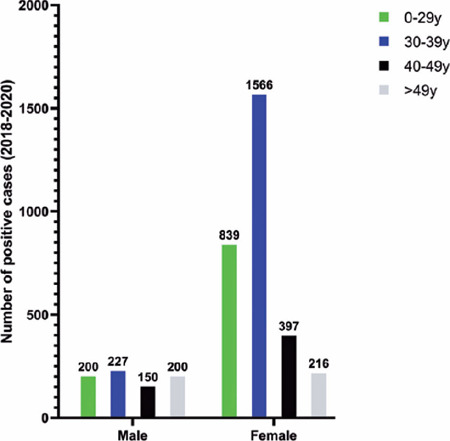
Distribution of IgM positivity for toxoplasmosis over 3 years, in men and women.

In women, the greatest number of positive cases (1,566) during the study occurred in the age group of 30-39 years (51.9%). There was no significant difference in the distribution of cases in relation to age in men (Figure 1).

### Description of toxoplasmosis foodborne outbreak (2019)

From February to May 2019, two clusters (A, B) involving 83 cases were investigated.

A total of 31 patients out of 83 (37.3%) were identified in the outbreak (positive IgM, with systemic symptoms) and underwent eye examination. Seventeen were from event A (Arabic restaurant) and 14 from event B (barbecue party). A patient from event A, previously with no ocular lesion, returned 14 months after the initial systemic disease with a retinal lesion consistent with ocular toxoplasmosis.

### 1 - Outbreak A (Arabic food restaurant)

This outbreak began in an Arabic food restaurant and lasted from February to May 2019. At least 47 people were infected. Most of the cases occurred in women (53%) aged 20-49 years (average 31 years) and 91.5% presented with a compatible clinical manifestation. All were positive for toxoplasmosis IgM and 97.8% (46/47) required medical management. Approximately 1,000 people attended the restaurant during the outbreak.

The average incubation period was 8 days (range 3-16 days). 57.4% consumed “falafel or special kebab” (vegetarian dish) and 31.9% consumed the “special kaftan kebab” (beef). All the dishes were accompanied by salad and tabbouleh. The predominant symptoms were fever (91.5%), headache (87.2%), and myalgia (80.9%).

Of 47 cases, 35% underwent ophthalmological evaluation. Four cases (23.5%) were diagnosed with focal re-tinochoroiditis, three of them during the first 3 months and one patient presented 14 months later with ocular symptoms. One patient had a macular lesion, with a loss of central vision in one eye after 10 weeks. The number of cases with eye lesions was therefore at least 4 (8.5%).

### Case 1, cluster A:

**Figure 2:**
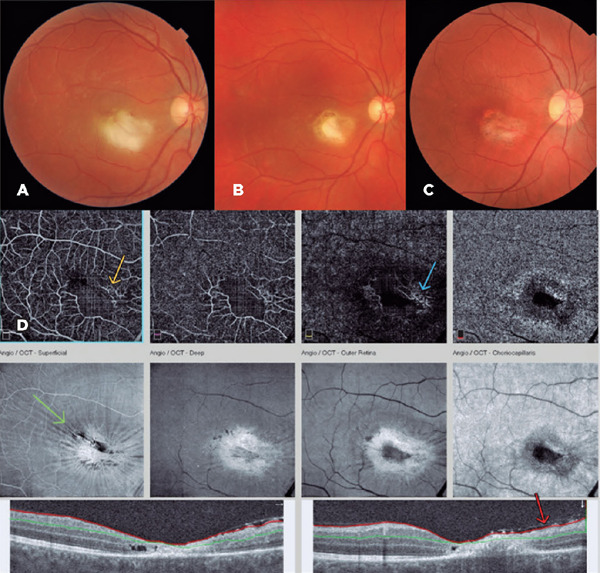
Fundus Photo, optical coherence tomography (OCT), and OCT-Angiography (OCT-A). (A) Retinochoroiditis 11 weeks after infection, (B) 14 days, and (C) 40 days after treatment with sulfamethoxazole/trimethoprim. (D) OCT-A and OCT after 2 months of treatment. On the OCT-A images, a vascular void adjacent to the foveal avascular zone is visible (yellow arrow). It is compatible with the lesion’s topography. The outer retina slabs show dilated vessels on the scar’s nasal borders that could suggest increased blood flow (blue arrow). The en-face slabs display a superficial concentric distortion suggestive of an epiretinal membrane and an atrophic scar (green arrow). OCT B-scans show retinal thinning due to disruption of the retinal layers and confirm the presence of an epiretinal membrane (red arrow).

A 32-year-old man attended a private eye clinic complaining of floaters and reduced vision (20/400) in the right eye. After ophthalmological evaluation, a diagnosis of retinochoroiditis was made (Figure 2). Serology for toxoplasmosis was requested (ELISA IgG+ and IgM+), and he was treated with sulfamethoxazole/trimethoprim and prednisone. The patient confirmed attending restaurant A 2.5 months before the eye complaints began. He developed fever, malaise, headache, and myalgia 5 days later and was seen twice by a general practitioner. In a referral hospital, he received medication for pain and fever, with dengue as the main diagnostic hypothesis. He did not undergo specific serology or receive specific treatment for toxoplasmosise. At the end of his course of treatment, his final visual acuity was 20/70 in the affected eye.

### Case 2, cluster A:

**Figure 3:**
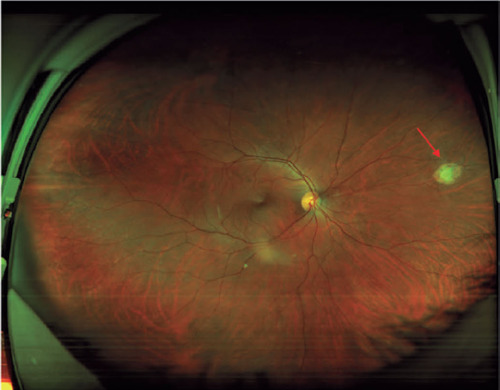
Wide field fundus color photography. The arrow shows a peripheral retinochoroiditis lesion 3 months after the onset of systemic acute toxoplasmosis.

A 32-year-old vegetarian woman developed a severe headache, myalgia, and fever. After 8 days, she was hospitalized in an intensive care unit with symptoms of meningitis. IgG and IgM (ELISA) for toxoplasmosis was positive but she did not receive specific systemic treatment for toxoplasmosis. Three months later, an ophthalmological evaluation confirmed the presence of active retinochoroiditis in the right eye (Figure 3). The final visual acuity was 20/20 in both eyes.

### Case 3, cluster A:

**Figure 4:**
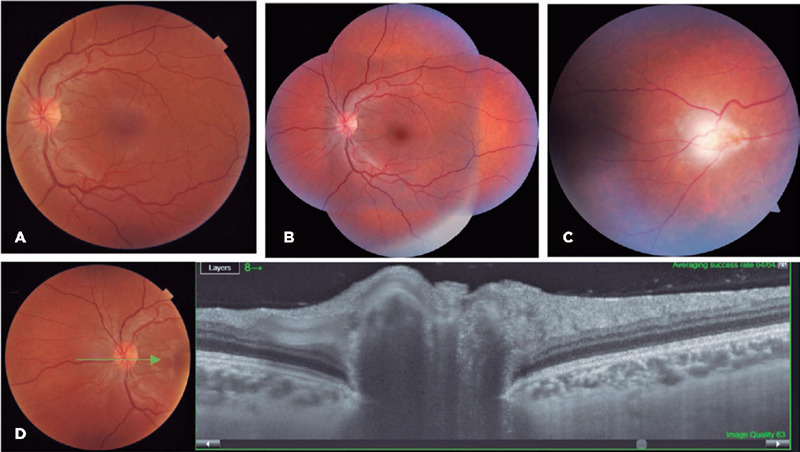
(A) Fundus photography shows optic nerve hyperemia in the left eye. (B) Wide field image. (C) Nasal peripheral active retino-choroiditis lesion in the right eye, with adjacent vasculitis. (D) OCT shows a change in the optic disk shape, suggestive of disk swelling.

A 24-year-old woman confirmed attending the Arabic restaurant (on the same day as case 2 and one day before case 4) where she consumed only vegetarian food. After 8 days she developed symptoms of headache, myalgia, adenomegaly, malaise, and fever. After 10 days without improvement, serology for toxoplasmosis showed positive IgG and IgM (ELISA), but she was not treated. After 2 months, the patient complained of floaters. Ophthalmological evaluation showed vitreous cells and optic nerve hyperemia in both eyes, and an active retinochoroiditis lesion in her left eye. OCT showed signs of inflammation of the optic disc (Figure 4).

### Case 4, cluster A:

A 26-year-old woman confirmed having been at restaurant A one day after patient 3, and consumed “special kaftan kebab” (beef) and tabbouleh. After 8 days she noticed systemic symptoms similar to the previous cases. Two days later, she was hospitalized with suspected dengue and meningitis. After 20 days of symptoms, she was tested for toxoplasmosis and the ELISA test (IgM and IgG) was positive. Treatment with sulfamethoxazole/trimethoprim was started, but the patient developed severe glottis edema after one day and the treatment was withdrawn.

In June 2020, 14 months later, an active peripheral retinochoroiditis in the right eye was diagnosed. Table 2 summarizes the epidemiological and clinical aspects of the cases with retinochoroiditis.

**Table 2:** Clinical features of the acute toxoplasmosis cases with retinochoroiditis

	Date (2019)	Main Course	Systemic Treatment during the acute disease	Incubation period days	Onset Retinochoroiditis
Case 1	Feb/24	Kafta (beef)	No	5 days	2 ½ months
Case 2	April/05	Falafel Kebab (vegetarian)	No	8 days	3 months
Case 3	April/05	Falafel Kebab (vegetarian)	No	8 days	2 months
Case 4	April/06	Kafta (beef)	No	8 days	14 months

### 2 - Cluster B (barbecue party)

From 70 adults that attended a barbecue party, 36 cases of acute toxoplasmosis were diagnosed, mostly in women (68.5%). The average incubation period was 5 days (range 4-9 days). The main symptoms were fever (95%), headache (68.2%), and myalgia (72.7%). Hospitalization was required in 50% of cases, and of these, 61% (11) received systemic treatment for toxoplasmosis, most of them with sulfadiazine-pyrimethamine (54.5%).

Forty-five items were served on the barbecue, including meat (beef, chicken, pork, and sausages), vegetable mayonnaise, salads, garnishes, sweets, and drinks. Twelve patients (33%) denied ingestion of meat.

In total, 14 of the 36 infected patients underwent eye examinations (38.9%) 3 months after the infection, which were normal. All 36 denied ocular symptoms after 2 years.

### 3 - Cluster A and Cluster B: Main Differences

The differences between the two clusters regarding the presence of retinochoroiditis (4 cases in cluster A and no cases in cluster B), was not significant (p=0.14). Of the total number of patients that underwent ophthal-mologic examination, with and without treatment, we observed that 100% of the cases with ocular lesions were not treated, while 63% of the cases without lesions received treatment for toxoplasmosis. This difference was statistically significant (p=0.03), as shown in table 3.

**Table 3:** Distribution of cases that underwent ophthalmologic exam, treatment status, and presence of retinochoroiditis, in both clusters

	Systemic Treatment	No Systemic Treatment	Total	p-value
RC lesion	0	4 (100%)	4	p<0.001
Without RC lesion	17 (63%)	10 (37%)	27	
Cluster A	6 (35%)	11 (65%)	17	p=0.032
Cluster B	11 (78.6%)	3 (21.4%)	14	

In patients undergoing ophthalmologic evaluation, in cluster A 35% of cases were treated in the acute phase of the disease, while in cluster B 78.6% of cases were treated. This difference was statistically significant (p=0.03).

## DISCUSSION

Data collected from outbreaks of clinical toxoplasmosis in humans can provide useful information concerning the infectious stage (oocyst versus tissue cysts), incubation period, and clinical spectrum^(6)^.

During the outbreak, laboratory data indicated a higher percentage of positive cases in both genders (p<0.01), but greater in men. Women were more affected in the outbreak, representing 59% of the cases, 25 in cluster A and 24 in cluster B. In women, most positive cases (laboratory data) occurred in the age group of 30-39 years (51.9%) (Figure 1). In Brazil, serology for toxoplasmosis is recommended in prenatal care, and should be repeated in the second and third trimester in vulnerable pregnant women, which may account for the higher rate of testing in women. Despite greater testing in women, we observed that during the outbreak period (Feb-May, 2019) there was an increase in laboratory seropositivity in men; the reason is unclear, but it may be due to differences in habits and hygiene in food consumption between men and women.

The comparison of positive IgM serology for 2018, 2019, and 2020 showed a 73.2% increase in 2019 compared to the previous year and 145.4% compared to the following year. As the study period was short, a longer follow-up time is needed for a better understanding of the changes in the frequency of the disease.

The age group with the greatest increase in the number of cases was between 45 and 60 years. It is possible that the disease is more symptomatic in this group and thus more symptomatic people were tested. Some studies also indicate a greater severity of ocular toxoplasmosis at this age^(7)^.

The patients’ signs and symptoms in this outbreak differed in multiple ways from the expected. Although infection in immunocompetent persons is usually asymptomatic or mildly symptomatic^(2)^, 98% of the patients in event A and 50% in event B in this outbreak needed medical assistance. Previous studies have shown that, in intermediate hosts, infection by oocysts is considered more clinically severe than infections caused by cysts^(8)^. The symptomatology could also be related to the amount of ingested food, parasite burden, or virulence of the infecting strains^(9)^.

In event A most of the patients did not consume meat, 57.4% of them consumed only vegetarian dishes such as salad and tabbouleh. The incubation period of 8 and 5 days (Event A and Event B), may suggest the ingestion of oocysts. The literature describes an incubation period from 10 to 23 days for toxoplasmosis after eating undercooked meat with cysts, and from 5 to 20 days after ingesting oocysts^(10)^. Drinking water contaminated with oocysts was responsible for the largest published outbreak of toxoplasmosis in 2018, in the city of Santa Maria, Brazil^(11)^.

In this study, 23.5% of the total cases that underwent ophthalmologic examination in outbreak A had retino-choroiditis lesions, 75% occurring in the first 3 months. The ocular lesions were more frequent between 2 and 3 months after the systemic disease. One of the cases presented with significant visual damage associated with macular lesions. All cases presented with systemic symptoms, with an initial diagnosis of dengue or sinusitis. Despite severe systemic disease, none of the cases with eye lesions received specific treatment in the acute phase of the disease. The greater number of cases treated in the acute phase in cluster B may be due to the prior knowledge of cluster A, detected by the surveillance system; this may have alerted clinicians to the diagnosis and encouraged systemic treatment. We emphasize the importance of guidance regarding the need for an ophthalmological examination in case of eye complaints after the disease.

Among patients who underwent ophthalmologic evaluation, a higher occurrence of retinochoroiditis was observed in untreated cases of acute toxoplasmosis, but the small sample size of this study precludes any conclusion about this. In a study from Erechim, Brazil, the records of 302 patients with serologic evidence of recent T gondii infection were analyzed. Antiparasitic treatment during the acute disease was associated with less ocular involvement^(12)^. Ophthalmological evaluation was performed in only 38% of the cases of this outbreak, therefore, we cannot estimate the actual prevalence of eye damage, as many cases may have been asymptomatic and were not evaluated.

Our findings indicate that the source of the outbreak was possibly vegetable contamination. Vegetables can transmit the infection in a similar way as meat. Greater attention should be paid to the disinfection of vegetables and fruits, and the quality of water used for drinking and irrigation, since water can be a contamination route for food^(13)^. The small number of cases is an important limiting factor in this study. More robust data are needed for further conclusions.

Our results show a high prevalence of toxoplasmosis in São Paulo, Brazil during an outbreak period and a drop in the number of tests during the COVID-19 pandemic^(13)^. The cases identified in the 2019 outbreak were most likely the result of some local condition in that period. We confirm the need to implement effective means for the prevention, diagnosis, and treatment of the disease. This may involve raising food hygiene awareness in the population and greater quality control of meat and water. A better understanding of the need for treatment of symptomatic patients in the acute phase of the disease is necessary.
